# "The Hospital" Nursing Mirror

**Published:** 1898-11-12

**Authors:** 


### "The Hospital" Nursing Mirror.

BEING THE NURSING SECTION OF "THE HOSPITAL."

[Contributions for this Section of "THE HOSPITAL" should be addressed to the Editor, THE HOSPITAL, 28 & 29, Southampton Street, Strand, London, W.C., and should have the word "Nursing" plainly written in left-hand top corner of the envelope.]

### News from the Nursing World.

### PRINCESS CHRISTIAN'S VISIT TO BIRKENHEAD.

ON the 3rd inst. the Princess Christian, who has been staying at Knowsley, in the neighbourhood of Birkenhead, visited the latter place to attend a public meeting at the Town Hall, and to receive a purse of money in aid of the District Nursing Association. Her Royal Highness was accompanied by the Princess Victoria, Lord Derby, Baroness Egloffstein, and Major Martin, and was received by the Mayor and Mayoress. Amongst those present were the Duke and Duchess of Westminster, Lady Margaret Ismay, the Bishop of Shrewsbury, Sir William and Lady Forwood, Sir Edward and Lady Russel. The Master of St. Katherine's Royal Hospital (the Rev. A. L. P. Peile) addressed the meeting at length, explaining the object, origin, and working of Queen Victoria's Jubilee Institute for Nurses, of which he is the secretary. The Hon. Secretary of the Birkenhead District Nursing Association gave an account of the local work; and Lady Margaret Ismay (one of the committee) described the extreme poverty in some of the homes amongst which the nurses worked, and asked the children of the neighbourhood to try and raise £500 for the purpose of securing a free bed at the Children's Convalescent Home, West Kirby. She also suggested that the birthday of the Princess Christian (the 25th of May) and Lord Mayor's Day (the 9th of November) should be kept as "pound days," when gifts in kind for the use of the society should be received. The Princess Christian has given her consent to the cot bearing her name as a memorial of her visit. The purse which was presented on this occasion contained £140, which is to be devoted to the building fund of the new home. One promise of £100 was made, contingent on an equal sum being subscribed shortly. After the usual votes of thanks, the Princess took tea in the Mayor's Parlour, which was decorated with the Della Robbia ware, and afterwards returned to Knowsley with Lord Derby.

### DISTINCTION FOR AUSTRIAN NURSES.

THE medals of the new Elizabethan Order, instituted for the reward of merit by the Emperor of Austria in memory of his wife, have been awarded to those engaged in combating the recent outbreak of plague in Vienna. Dr. Rudolph Pock has received the Knight's Cross, and Nurse Hochecker and the Sisters of Mercy the Elizabeth medal.

### A PLEASANT EVENING.

A VERY pleasant evening was spent at the North-Eastern Hospital for Children in the Hackney Road on Thursday evening by a numerous company of those interested in the institution, who had come together to celebrate the acquisition of a Röntgen ray apparatus, which had been presented by the Duke of Newcastle. Mr. J. Lister Godlee, the treasurer, and Mrs. Godlee received the guests, amongst whom were the Duke of Newcastle and the Bishop of Stepney. Tea, coffee, and light refreshments were served in the out-patients' department, which had been prettily arranged for the occasion, and where an excellent band played selections of music. In one room the wonders of cinematograph were displayed, and in another those of the Röntgen rays. The guests passed in small parties through the two wards containing in all 57 beds, and saw the sick children, for the most part peacefully slumbering in their comfortable, cheery-looking cots. Interested nurses spared no pains to make the visit instructive. There are many little Jews and Jewesses in the wards, noteworthy for the evidences they afford when they arrive of their parents' dislike to soap and water, and of the deep family affection that exists amongst them. The fathers are especially devoted to their little ones, frequently accompanying them to the out-patient department on Saturdays. Negotiations are in progress which, it is hoped, will give the hospital a site whereon to build additional wards. A sum of £25,000 will be needed for this purpose, and the contributions already in hand reach only £15,000.

### NURSES' NATIONAL TOTAL ABSTINENCE LEAGUE.

LAST year a branch of the Women's Total Abstinence Union was formed for the mutual help of trained nurses on this point. It was named the Nurses' Total Abstinence Union, and Miss Holland, 4, Ludgate Hill, E.C., was elected the secretary. The minimum subscription is one shilling annually, and *Wings*, the official organ of the parent society, is sent free monthly to every member. The Hon. Mrs. Eliot Yorke is the president, and one of the vice-presidents is Miss Orme, the late matron of the London Temperance Hospital. Six members of the committee are nurses, and meetings are held four times a year. All who know the many temptations to take stimulants to which nurses are exposed, and their baleful effect when once the dividing line between strict temperance and a little too much is passed, will be glad to hear of the success of a society that strikes at the root of the evil. It is not everyone that can go so far as total abstinence, but there are many who can, and some who must do so if they are to pass through life in honour and happiness. Therefore, whatever one may deem right for oneself, let us hold out an encouraging hand to those who think it their duty to be total abstainers either for their own sake or for that of others.

### ROYAL NATIONAL PENSION FUND FOR NURSES.

THE Secretary of the Pension Fund requests us to inform policy-holders who require information as to the bonus additions now being distributed, that it is unnecessary to call at the office in person, and it will greatly help the officials if they will kindly send written inquiries. The Secretary desires to take this opportunity to thank the large number of nurses who have written letters of appreciation at the present time.

### WOMEN'S HELP TO WOMEN.

Of all social work, that dealing with the redemption of women from a life of degradation appeals most strongly to any who sympathise with the progress of the race or with the happiness of the individual. In fact, no one can afford to neglect it, though as yet, in proportion to magnitude of the evil, so little has been done that it may be said with truth that only the outside of the canker is being salved. The Female Mission to the Fallen, however, is an agency that has been quietly at work for the last forty years, its headquarters being at 32, Charing Cross, Strand. The president is the Marquis of Ailsa; the patron the Archbishop of Canterbury. Its missionaries visit the streets at night, and invite the women to come back to the right path. In the course of its existence it has succeeded in assisting in different ways some 31,876 women, and last year £3,700—of which sum £2,500 have still to be raised—were spent. A special appeal has therefore been issued by the president. Mission houses have been established in North, South, East, and West London, to afford temporary shelter to the passing guests, whilst arrangements are in progress for permanently helping them.

### A "NURSE" BALLAD.

One of the prettiest poems that we have seen in praise of the "Nurse" appears in the *London Hospital Gazette* for October, 1898, under that title. It is sung by one who says "I'm a wastrel—I'm a bad'un," and tells how he'd "been knifed" and brought into the hospital, and whilst the doctors were "shoving their stitches through him," and he was swearing, "he clapped his eye on 'er, and it stopped 'im in a minute." He then goes on to describe how he watched her when he was coming to his senses, and the things "a bit confoosing" became clearer; and how "'er smile, twas like the dawning—Gawd's angels must have taught her 'Ow to smile like that on blokes of common clay." And lastly, how when sleeping later in a "doss" house, and awakened by some row, he calls out, "Nurse! just like I was in pain," and then falls asleep and "dreams of 'er again."

### THE WORRY OF BADGES.

To a certain class of minds there is only one remedy to the evils which the trained nurse has to face, and that is she must wear a badge. The uniform may be copied and abused, but the nurse is all right if she can but add to that uniform a badge. Incompetent nurses will be banished for ever, and the ideal nurse sail through life as on an untroubled sea if she but pin to her breast the magic badge. There are, however, a few difficulties that hinder the dawn of these halcyon days. Who is to give the badge? Trained nurses who desire to buy it for a small sum are in exactly the same position as those who are not nurses at all. Anyone can buy a badge, or, failing that, borrow or steal one; and who would be the wiser, or who could put a stop to such practices? Then, after a nurse has her badge, who is going to guarantee that she will not disgrace it? No one is all bad, neither is anyone all good; and, alas! there are as many chances of failure amongst trained nurses as in any other section of the community. This being the case, will the wearing of a badge save them, or will they bring discredit on the symbol, and cause others to abandon it? A nurse desiring a good reputation had better by far so regulate her own life as to win the approbation, first of her own conscience, secondly of her teachers, and, lastly, of her employers. Such a one needs no trade mark to single her out in the public gaze.

### THE JUBILEE NURSES OF CANADA.

Lady Aberdeen, before leaving Ottawa, took the chair at a meeting of the Board of Governors of the Victorian Order of Nurses, which was inaugurated by her in commemoration of the Queen's Diamond Jubilee. The badges, approved by Her Majesty, had only been received from England on the previous day. They are of the same design as those worn by the "Queen's Nurses" in England, with the additional superscription, "Victorian Order of Nurses for Canada." Lady Aberdeen, by unanimous request of the Board of Governors, retains the position of president of the Order, and the Countess of Minto has been invited to become honorary president; Mr. Cox, Hon. Senator of Toronto, and Mr. Drummond, Hon. Senator of Montreal, were elected vice-presidents; the chief Lady Superintendent and the District Superintendents of Ottawa, Montreal, Toronto, Halifax, and Klondyke were admitted to full membership; and the nurses who had successfully accomplished their district training were formally recognised as belonging to the Order, Lady Aberdeen herself bestowing the distinguishing badges.

### OUR CLOTHING DISTRIBUTION FUND.

It is with great pleasure that we acknowledge the receipt of our first parcel of clothing. Nurse Hillman, of Charmouth, Dorset, has sent four charming little pink frocks, three pinafores, and two vests. This first-fruits is most cheering, and we look forward to a continual stream of useful gifts during the next five weeks.

### A YANKEE PLAN.

Brooklyn, New York, has set agoing a novel scheme. A nursery has been arranged in the basement of the church, at which mothers may leave their infants whilst they attend divine service. It seems an excellent plan in many ways, but the opportunities for the interchange of undesirable microbes is too great to render it attractive to careful mothers, although it is not impossible that there might be mothers who would forget to claim their offspring at the close of the proceedings.

### SHORT ITEMS.

Miss Anna McGee, who was appointed acting assistant surgeon in the army of the United States, receives the pay and quarters of and ranks with second lieutenant. She has chosen the thirty nurses from the Brooklyn and Manhattan Training Schools.—A concert was held recently in the Town Hall, Rugby, in aid of the District Nursing Association, when a long and enjoyable programme, occupying a couple of hours, was given. The President of the Association, who arranged it, had reason to be pleased with the results of his efforts.—Some misconception having arisen with respect to Sunderland Nursing Institute and the new home, Victoria House, just opened, we take the opportunity of stating that the Sunderland Nursing Institute and Home for Trained Nurses still occupies the premises in Borough Road bought by the committee in 1889. The work handed over to the Queen's nurses in January last was the district branch begun nine years ago, and it is for this section of the work that the Victoria Home is to serve.

### Notes on Gynæcological Nursing.

By ARTHUR E. GILES, M.D., B.Sc., F.R.C.S., Assistant Surgeon Chelsea Hospital for Women.

### VI.—NURSING OF PATIENTS AFTER OPERATION (*continued*).

It is very important that the gynæcological nurse should be in a position to recognise the early symptoms of the dangers to which a patient is liable after abdominal section. Immediately after operation there are dangers attendant on the anæsthesia, notably the risk of choking from mucus or vomited matter obstructing the larynx. To prevent this the patient should lie with her head on one side, the lower jaw should be gently held forward, and the mouth kept a little open. If a rattling sound in the breathing should indicate the presence of mucus the mouth should be swabbed out with a handkerchief, or better still, with a small cotton wool swab on a holder.

SHOCK.—This is indicated by a small thready pulse which can hardly be felt, a sub-normal temperature, coldness of the extremities, and a cold sweat on the forehead. It is the signal for increased warmth by hot-water bottles and blankets, and stimulants may be given under the doctor's directions. In case of sickness a nutrient enema of brandy, 1 oz., and beef-tea, 3 oz., answers very well. The recovery from shock is indicated by returning warmth and a fuller pulse; the reaction may send the temperature up to 100 deg. F., or higher.

HÆMORRHAGE.—The signs of internal hæmorrhage are similar to those of shock, whilst in addition the pulse is very rapid, and the patient is restless, throwing her arms and head about, and with a sighing respiration. It is very seldom that internal hæmorrhage supervenes until after the patient has recovered from shock, but it may come on at any time within the first few days after operation. It is most important that the nurse should send for assistance at the earliest possible moment after she has noticed signs of hæmorrhage, since the danger increases every minute, especially in bad cases. It is dangerous to give stimulants when internal hæmorrhage is going on.

SEPTICÆMIA.—The first symptoms of blood-poisoning may come on from the second to the fourth day after operation. The temperature runs up to 103 deg. or 104 deg. F. The rise is sometimes preceded by a rigor. The patient's tongue becomes dry, and coated at first with a white fur; later it is brown, and the breath is often offensive. Pain is often complained of, but is not invariable. An almost constant feature of septic peritonitis is abdominal distension, which may proceed to an extreme degree, and sooner or later vomiting comes on. The patient may become delirious, but this also is not constant. Distension is often one of the earliest signs, and when accompanied by high temperature is always a cause for anxiety.

RESULTS OF ACCIDENTS DURING OPERATION.—Occasionally during an abdominal operation the bowel is wounded, and this may result in a fæcal fistula. The nurse will then notice the presence of fæcal discharge in the dressings, and she should give the surgeon immediate notice of the fact. A wound of the bladder or ureter may similarly result in a urinary fistula, which can be recognised by the urinous smell in the dressings. The same accident may occur after some vaginal operations. The nurse must distinguish this from mere incontinence of urine.

### NURSING AFTER VAGINAL OPERATIONS.

1. CURETTING, TRACHELORRHAPHY, &c. — After these operations the nurse has little to do beyond douching and the passing of the catheter in certain cases. We must repeat that it is always undesirable to pass a catheter if it can be avoided, for besides the discomfort of it there is always the risk of cystitis, unless the most rigid precautions are taken. Many a patient has recovered satisfactorily from the actual operation, and has had her convalescence delayed by this troublesome complication. This is, of course, most trying to all concerned. Douching is commonly not required for the first twenty-four or forty-eight hours. I may refer my readers to the third lecture of this series (see THE HOSPITAL, September 17th, 1898, "Nursing Mirror," p. 211) for some practical hints in the matter of douching.

2. EXTERNAL OPERATIONS. — There are a few of these where a dressing is applied, *e.g.*, perineorrhaphy, dissection of cysts or abscesses of the Bartholinian glands, removal of tumours of the vulva, &c. Here the difficulty is to keep the dressings clean, and free from discharges from the bladder, vagina, and rectum. Consequently, the dressings may require changing pretty often. Much depends on this point, for once the dressings become contaminated the wound is apt to become infected, when it may suppurate and break down instead of healing by first intention. Even if this should not occur, a stitch-abscess may form which would delay healing, perhaps for several weeks. Although circumstances render it impossible to maintain as rigid an asepsis here as in other parts of the body, no effort should be spared to make the conditions as favourable as possible. Broadly speaking, it should be remembered that aseptic and antiseptic precautions are just as necessary in the changing of dressings as in the performance of an operation.

3. VAGINAL HYSTERECTOMY.—This is the most serious of vaginal operations; but apart from such nursing as every operation case requires, there is usually little for the nurse to do for the first few days. The catheter is very often necessary, and in its use precautions must be redoubled, for the bladder is so closely involved in this operation that it is much less tolerant of interference than in ordinary cases. The vagina is often packed with tampons or gauze at the close of the operation, and it may sometimes be the nurse's duty to remove them. This must be done with the greatest care, for there are often ligatures left in the vagina, dragging upon which might result in serious hæmorrhage. Some surgeons employ clamps, which are allowed to remain attached for forty-eight hours after operation. In the handling of these similar care must be exercised; for instance, when the patient is put back to bed it should be one nurse's duty to lift the clamps when the patient is lifted, and when the patient is in bed the handles of the clamps should be supported on a small cushion, or on a pad of cotton wool. Douching is generally begun five or six days after the operation. There is generally a hæmorrhagic discharge for a week or ten days after operation. After the simpler operation of curetting, such a discharge may occur for a few days or a week. It is not necessary to keep a patient long in the dorsal position after vaginal hysterectomy; indeed, she may be allowed to turn on her side on the evening of the first day, except when clamps have been used; and providing that the pulse be good, it is often an advantage for her to be propped up in bed for a short time occasionally a few days after the operation. In conclusion, let me remind the nurse that, as in the preparation for operations, so in their after treatment the successful issue of the case, and sometimes the life of the patient, may depend on the minute and conscientious care with which the nurse performs her duties.

### Wants and Workers.

[THE attention of correspondents is directed to the fact that "Helps in Sickness and to Health" (Scientific Press, 28 & 29, Southampton Street, Strand, London, W.C.) will enable them promptly to find the most suitable accommodation for difficult or special cases.]

MRS. GILLETT, North Newington, Banbury, would be glad to hear of a secondhand water bed for sale, to be used in district nursing.

### Antiseptics for Nurses.

BY A MEDICAL WOMAN.

XXII.—DISINFECTION BY HEAT—(*continued*).

THE reasons why steam or moist heat is superior to dry for disinfecting have been shown by Dr. Parsons to be the following: In steam there is a greater amount of latent heat than in dry, and it requires nearly 1,000 times as much heat to change 1 lb. of water at 212 deg. F. into steam as it does to raise the same amount of water 1 deg., or from 211 deg. F. to 212 deg. F., and conversely a corresponding amount of heat is liberated when 1 lb. of steam at 212 deg. F. is condensed with water at 212 deg. F. When an object is heated by being placed in hot, dry air, not only is no latent heat yielded up to it by the air, but, on the other hand, before the object can attain the temperature of 212 deg. F., any water which it may contain (and all textile fabrics, even though they have been dried at ordinary temperatures, retain a quantity of hygroscopic moisture) must be evaporated. In this evaporation heat passes into the latent form, and the attainment of the required temperature is therefore delayed.

When steam penetrates into the interstices of a cold body, it undergoes condensation in imparting its latent heat, as aforesaid, to the body. When condensed into water it occupies only a very small fraction of its former volume. To fill the vacuum thus formed more steam presses forward, and in its turn yields up its heat and becomes condensed, and so on until the whole mass of the cold body has been penetrated. On the other hand, hot air, when yielding up its heat, undergoes some slight contraction in volume, it is true, but only to a very small extent as compared with that which is undergone by steam in condensing into water.

The heat which is evolved in the moistening of a dry porous substance is another source of advantage on the side of steam. Pouillet, in his "Annales de Chimie," has shown that it is a general law: (1) That when a liquid wets a solid there is a disengagement of heat; (2) that when a solid absorbs a liquid there is a disengagement of heat. The amount of heat liberated in the incorporation of a liquid into the substance of a solid is much greater than that liberated by mere superficial wetting; this is owing to multiplication of points of contact. In proportion as a wetted substance is finely divided the number of particles which are moistened and take part in the evolution of heat is multiplied, while the number is diminished of those which, not being wetted, do not give out heat, but absorb it at the expense of others. Hence porous organic substances, as being most finely divided, are those which, in moistening, evolve most heat; of course, chemical reactions, like the slaking of lime are excluded. If a clinical thermometer be wrapped up in a few turns of flannel which has recently been highly dried before the fire and then cooled, and the roll be held in the mouth and the expired breath forced through it, a temperature considerably over that of the breath will be found to be registered by the thermometer. In such an experiment Dr. Parsons found the thermometer to reach 110·6 deg. F., whilst the temperature of the breath, as taken by the same thermometer, was only 99·2 deg. F. If the flannel, in which the bulb of the thermometer is wrapped, has been dried merely in the air at the ordinary temperature and humidity, a rise of temperature above that of the breath will still be obtained, but of less extent than in the previous case. If, on the other hand, the flannel is damp, no elevation at all will be obtained.

The specific heat of steam is greater than that of air in the proportion of about 5 to 4, that is to say, 4 volumes of steam in falling 1 deg. F. without condensing into water yield up as much heat to other objects as 5 volumes of air at the same temperature yield. The rate at which steam will diffuse into air, as compared with that at which air will diffuse into steam at the same temperature, is as about 5 to 4, hence, excluding the influence of differences of temperature, steam would diffuse into air with a rapidity one-fourth greater than air would. The penetration of heat, in the form of steam, is aided by pressure. By relaxing the pressure of steam, and then re-applying the pressure from time to time the penetration power of the steam is greatly increased. It has further been shown that a pressure of steam equal to 15 lbs. to the square inch above that of the atmosphere compresses the air in the interstices of the material to half its original volume, and thus the space originally occupied by cold air becomes partially occupied by steam. Thus steam partly mixed with air occupies the interstices near the surface, whilst air occupies those in the centre. When equilibrium of pressure has been obtained, the interchange of the air and steam particles and of temperature takes place comparatively slowly. When, however, the pressure is relaxed, the air expands again to its original volume, and thus becomes mixed with the steam, and when the pressure is reapplied the steam is forced still further into the interstices.

Mr. Defries has recently shown that superheated steam is not so efficacious for disinfecting purposes as is saturated steam, but that the effect of the two in disinfection is quite different, and explains as follows: The terms superheated and saturated are used in the sense that at a given pressure steam is saturated when it is of the temperature at which under that pressure it can condense, or at which water under the same pressure can become steam, whereas it is spoken of as superheated when it is of a higher temperature. At the ordinary atmospheric pressure, for instance, steam is saturated at 212 deg. F. Having now considered the main principles of disinfection by heat, we will describe some of the best known apparatus. One of the most widely used of these is the Washington Lyons' steam apparatus, which consists of a large, strong iron chamber with double walls of boiler plate, and a tightly-fitting door at either end, which shuts against an india-rubber collar, and is secured by vice handles so as to form a steam-tight joint. It is also hung on hinges, and its weight, when swung open, is borne by a castor running on a curved rail. By means of a steam pipe steam from a boiler can be admitted into either the hollow casing or the interior of the chamber; another pipe serves to let off the steam when no longer required, and there is also a means of escape for condensed water, which may accumulate in the casing. The casing and chamber are each provided with a safety valve and pressure gauge, by which the pressure of steam, and hence the temperature, can at any moment be seen, the degrees of temperature being marked on a dial and indicated by a needle. In using the apparatus, steam is first turned on so as to heat the casing or jacket, and in this way the walls are warmed, and it is advisable to let the steam circulate for some time in the jacket, so that when admitted into the chamber there will be little condensation and wetting. The articles to be disinfected are now put into the chamber and the door closed, and are thus left for some time, whilst steam is admitted so as to become thoroughly heated, so as to avoid condensation. Finally steam is allowed to enter the chamber until the gauge shows it has reached the desired temperature. When disinfection is completed, the steam is first shut off from the chamber, but allowed to circulate in the jacket so as to dry the articles, and on ultimately opening the doors and removing the clothes they will be found to be quite dry.

### Lectures on Medical Relief.

UNDER THE AUSPICES OF THE CHARITY ORGANISATION SOCIETY.

### THE DISPENSARY.

THREE of this series of eight lectures have now been given on Friday afternoons at the Portman Rooms. The first, by Mr. Loch, was of an introductory character, and treated the subject from a broad and general point of view. In the second lecture Dr. Hawkes traced the history of dispensaries, institutions carrying out the functions of a hospital out-patient department, with this difference, that those patients too ill to visit the dispensary were treated at their own homes. The idea of dispensaries originated at the end of the last century, when several philanthropic medical men began to see gratuitously at their own houses, at certain hours, any of the poor who wished to consult them. This custom presently caused adverse criticism on the part of the College of Physicians, as not consonant with the dignity of the medical profession, and its consequent abandonment led to the formation of medical and lay committees, who hired a house and appointed a staff, consisting of a physician and surgeon and a resident apothecary, by whom patients were seen gratuitously at the dispensary house or at their own homes; funds to carry on the work being raised by subscriptions or donations. From this small beginning sprang the whole dispensary system. The work done by these earliest institutions could hardly be too highly estimated. On them fell the chief brunt of all epidemics, such as cholera and small-pox. Dr. Hawkes went on to describe modern dispensaries, classifying them as: (1) Free dispensaries, (2) part pay dispensaries, (3) pay dispensaries proper, and (4) provident dispensaries. The latter, if properly and energetically conducted, had, Dr. Hawkes considered, a great future before them, the idea being one which appealed to the better class of workmen. Going on to consider the "letter" system, the lecturer spoke of its abuse and the carelessness shown in the wholesale distribution of letters. In New York, he said, the difficulty concerning inquiry into the circumstances of patients was in a measure overcome by the division of the city into districts, each with its own dispensary, patients not being allowed to attend any but that within their own area. Such a plan might not be impossible in London, by division into large districts, the hospital acting as a common centre. All cases would then be seen first at the dispensary, those needing confinement to bed being either treated at home or drafted into hospital, to which certain selected cases would be also sent for teaching purposes or for additional advice. Students would, as in Edinburgh, attend at the dispensaries to gain experience in the treatment of minor ailments. Attached to each dispensary would be an inquiry office, no person being eligible for treatment who lived outside the area, unless provided with a letter from a qualified medical man. There was much overlapping of medical charities in London at the present time. The help given to dispensary doctors by the trained district nurse was invaluable, not only in seeing that directions were carried out, but by the humanising and educating influence she exercised in the homes visited. Dr. Hawkes concluded by saying that too little was known of the value of the dispensary, partly because it was a purely local institution, for the most part supported by those amongst whom it carried on its useful work.

### THE HOSPITAL.

Anything that Mr. Clinton Dent has to say is sure to be worth hearing, if only for the amusing bits of sarcasm with which his remarks are invariably garnished. His lecture on "The Growth of Hospitals," given as the third of the series last Friday afternoon was interesting. Mr. Dent began by a description of hospitals as they existed in London at the close of the last and the beginning of the present century, drawing his illustrations from the notes of John Howard and other contemporary writers. He described the rough wooden floors, the wooden staircases, commenting on which John Howard made the pertinent remark that the stone stairs he had seen abroad were "more proper." The ventilation in the hospital of 100 years ago was very deficient; the wards were crowded, poorly lighted, very low, sometimes not more than 9 ft. 6 in. in height, with whitewashed walls and sanded floors. How little attention was paid to window opening might be gathered from a story related of a certain hospital where, it being found that patients' friends passed food in to them through the windows, the governors forthwith ordered them to be nailed up! Bedsteads were of wood, usually with testers, and well curtained up; patients lay parallel to the wall, and there was no space between the beds. In 1775 Samuel Sharp, surgeon to Guy's Hospital, wrote with approval of the iron bedsteads he had seen in Florence, and some forty years later they began to come into use. While recognising the defects of these hospitals, Mr. Dent reminded his hearers that it was in them that men like John Hunter, Ashley Cooper, and Benjamin Brodie lived and worked and succeeded. In fact, hospitals, then as now, were infinitely in advance of their time, and when public opinion suddenly exploded and matters improved they still led the way. Mr. Dent went on to speak of vaccination, the full value of which, he declared, the "conscientious objector will in a few years convince everybody"; and of the antiseptic system, the most beneficent discovery of the century. Nursing was sometimes spoken of as almost a new adjunct of hospital life, but it was not so. The "original" nurses, so to speak, were religious sisters, such as those of St. Vincent, and the Grey Sisters, women unquestionably animated with a very high ideal, which he could wish were more frequently to be found now. It was when these Sisters began to care for something more than their patients' bodies that they were succeeded by women of an inferior order. Speaking of hospital discipline, Mr. Dent mentioned a curious old ordinance of those days which directed the porter at a certain hospital (whose duties included that of seeing that the patients said their prayers), in the event of any person being found "a swearer or contemner of the matron, or if he "refuse to go to bed," that "him shall ye punish after once warning him in the stocks." Considering hospitals then and now, there was no change so remarkable as that which had taken place in the respective relations between the public and the hospital. In the early part of the century hospitals were probably only used by the poor and the very poor, and patients were never allowed to forget the fact that they were objects of charity. Now that they are better known things were very different. There could be no doubt that the better hospitals were built, drained, officered, and nursed, the more it tended to place patients who paid nothing and cost much on a level with those who paid more than they cost; the more benevolence was driven into the background, and a sort of right to prospective treatment was established. Hospitals had gradually changed from private institutions into public ones, being recognised as of public utility though receiving no state grant. Mr. Dent touched on the question of hospital abuse, and the scheme of a Central Hospital Board, in his view, a good idea, but presenting difficulties in practice, and likely to do little good unless in command of sufficient funds. The rate-paid hospital system might come, but it happily seemed far off. State subsidies would mean state control, and any such solution of present problems would be far less efficient, and would cost four or five times as much as the present system, besides seriously damaging medical education. Speaking of the increase of out-patients, the lecturer said he believed that the improved repute of hospitals was one reason for this, and he concluded by expressing his conviction that the whole object of hospitals should be to work with and not in antagonism to the general practitioner who works amongst the poor; and that no real change had taken place in the management and government of hospitals during the century, but that the general principles with which they were founded had been throughout loyally and unswervingly adhered to.

### A Nursing Hostel.

A VERY cosy nursing home is 33, Beaumont Street, of which Miss Meyrick is the lady superintendent. It is most conveniently situated, being close to Baker Street Station and to omnibus communication with Regent Circus. The lady superintendent was one of those who trained as nurse in early days. She was a probationer at St. Mary's Hospital, Paddington, in 1871, and matron of the Hospital for Gentlewomen in Harley Street up to 1890, and two years later she began her present work. The furnishing and arranging of the house show much taste, and that knack of imparting a homely feeling that contributes so much to the comfort of the inmates. The walls are papered with pretty, light papers and varnished; they are washed down every year and frequently in between times when occasion requires. The floors are polished and covered with rugs, the furniture is enamelled white, and the lady superintendent and the nurses give it a new coat of paint yearly, which makes it look like new. The bedsteads are brass, and are fitted with recent improvements, and in each room there is a roomy, comfortable couch. The nurses' accommodation is carefully arranged, and much thought is expended in securing their comfort and convenience. The uniform is of butcher-blue linen with white lace caps tied under the chin. The diet is good and well cooked, and neatly and prettily served. The house being a corner one there is an abundance of light, a necessity with so many operations. Electric light is used at night, and there are many sanitary conveniences not always to be found in such establishments.

### Where to Go.

STAFFORD HOUSE.—The winter sale of the Working Ladies' Guild will be held at Stafford House, by the kind permission of the Duke of Sutherland, on November 30th and December 1st. H.R.H. Princess Henry of Battenberg will open and preside at the sale on November 30th. First day, open from two to six o'clock; second day, twelve to six o'clock. Admission, 2s. 6d. and 1s. Tea and coffee stall.

THE DOWDESWELL GALLERIES.—These Galleries, at 160, New Bond Street, are now occupied by a most delightful collection of oil and water colour paintings by Mr. Oliver Hall, R.E. Mr. Hall confines himself to landscape, in the treatment of which his talent is most versatile. The poetic handling of trees is most noticeable, and many studies recall the work of some of the first masters of both French and English schools. The exhibition is well worthy of a visit.

### LONDON OPHTHALMIC HOSPITAL, MOORFIELDS.

ON November 9th a ball was given at the Empress Rooms, Royal Palace Hotel, Kensington, in aid of the funds of the above hospital. Herr Wurm's Blue Viennese Band was engaged.

### The Orphans' Home, Austral Street, West Square, S.E.

THREE excellent rules, but unusual in charitable institutions, obtain at the Orphans' Home, Austral Street. There is no begging, no voting, no debt, and judging by the report the result is excellent. From a small beginning thirty-three years ago Miss Sharman's work has grown until last year she received forty-nine children, the two youngest of whom had reached the mature age of two months. In connection with this home there are country branches at Overcliffe, Gravesend; Upper Grosvenor Road, Tunbridge Wells; a seaside branch at Mount Pleasant Road, Hastings; and an educational branch at St. Michael's Road, Stockwell. The London home is open to visitors on Thursday afternoons and the branch homes on any weekdays excepting Saturdays and Mondays. The girls are trained chiefly for domestic service, and Miss Sharman has more applications than she can supply. A second "old" girl has recently claimed the gold watch awarded to those who remain in their first places for seven years. There are many applications for admittance to the home that must be refused from lack of funds. As the cost of keeping an orphan for a year is only £15 sometimes friends of the work contribute the necessary money for an urgent case until a vacancy occurs.

### Minor Appointments.

PRESCOT UNION WORKHOUSE INFIRMARY.—The following Charge Nurses have been appointed at the above infirmary: Miss Frances Broadhead, who was trained at Manchester Royal Infirmary, and who has held appointments as charge nurse at the Carlisle Union Infirmary, and since has undertaken district nursing at Bournemouth and other places; Elizabeth M. Boulding and Mary H. Christie, who have both been charge nurses at Toxteth Park Union Infirmary; Rachel Taylor, who was trained at Salford Union Infirmary, and who has since been district nursing at Jedburgh.

ROYAL ALBERT EDWARD INFIRMARY, WIGAN.—Miss Lucy S. Snape has been appointed Sister of the female wards of this institution. She was trained at the Northern Hospital, Liverpool, to which, after a year's private work, she returned, and undertook temporary work as night superintendent for three months, and afterwards that of assistant matron.

THE MOTHERS' SEASIDE CONVALESCENT HOME, HERNE BAY.—Miss Alice M. Browne, formerly sister of Middle East Floor at the Dreadnought Hospital, Greenwich, has been appointed Lady Superintendent and Secretary of the above. Miss Browne was previously sister at the London Hospital, and holds the certificate of the L.O.S.

HACKNEY UNION INFIRMARY.—On the 19th ult. Miss Martha Sarah Davies was appointed staff nurse of the above. She was trained at the Children's Hospital, Paddington Green, and has worked at the St. John's Infirmary, Hampstead, and at St. Bartholomew's Home, Swanley, Kent.

HOLBORN UNION WORKHOUSE, MITCHAM, SURREY.—Miss Annie Youngs, who has been trained under the Meath Workhouse Attendants' Association, at St. Peter's Home, Kilburn, has been appointed Nurse here, and takes up her new appointment on the 12th inst.

OPHTHALMIC SCHOOL, HANWELL.—Miss Emeline D. Lynch, who has been superintendent nurse at the above school for the last two years, has now been appointed Matron in addition to her other duties.

THE COTTAGE HOSPITAL, FLEETWOOD.—Miss M. Storey, who was trained at the Leeds General Infirmary, and who has held various posts in the same, was appointed Matron of the above hospital on November 2nd, 1898.

### Training in the Provinces.

(*Continued from page* 48.)

### ANCOATS HOSPITAL, MANCHESTER (105 Beds).

### Terms of Training.

Candidates for training at the Ancoats Hospital should be between 23 and 30 years of age. If found satisfactory on the necessary personal interview, applicants are received for a month's trial, on the successful completion of which term of probation they enter upon three years' training. A certificate is granted at the satisfactory conclusion of the engagement. A sum of £5 is forfeited if a nurse breaks her engagement. No salary is given for the first year, payment beginning at £12 for the second year, and rising to £21 for the third year. Laundry is provided by the hospital, and indoor uniform is also supplied to the nurses. Sisters' salaries begin at £24 per annum, and rise to £32.

Night duty is usually taken for six months at a time. Lectures are given by members of the honorary medical staff and by the matron to the nurses during their training. A course of sick cookery is also included in the curriculum.

### Hours On and Off Duty.

The day nurses are on duty in the wards between 7.30 a.m. and 9 p.m., with two hours off duty daily, and time for meals. A half-day is given on alternate Sundays from 2 p.m. to 9 p.m., and frequently on other days as occasion arises. Two weeks' annual holiday is allowed to probationers, three to sisters, and an extra day for travelling. Night nurses' hours on duty are from 9 p.m. to 7.30 a.m.; they take their recreation either from 9.30 to 12.30 noon, or from 5 to 8.15 p.m. There is a library and a piano in the nurses' sitting-room for their use; they have also a tennis court.

### Meals.

The day nursing staff breakfast at 7 a.m.; lunch is at 10, dinner at 1.15 p,m., tea from 4 to 5, and supper at 9 p.m. No food allowances are given out to the nurses, except tea for lunch, daily supplies of all that is needed being sent to the dining.room, where all meals are served. Night nurses dine at 8 p.m., and breakfast at 8.30 a.m.

### MANCHESTER ROYAL INFIRMARY (298 Beds).

### Terms of Training.

A three years' course of training is offered to nurses at the Manchester Royal Infirmary, a certificate being granted after passing examinations and satisfactorily completing the engagement. Applicants should be between 25 and 30 years of age, and must be well educated. Personal application must be made to the lady superintendent of nurses at the hospital between 10 and 12 a.m. The agreement is to serve the hospital for three years, but after the first two probationers may be employed either in the infirmary, on the private nursing staff, or at the convalescent hospital, at the discretion of the matron. No premium is required from probationers, salary beginning the first year at £10, and rising to £15 and £18 for the second and third years respectively. After completing the third year, nurses are paid £20 per annum, rising £1 annually to a maximum of £25. Sisters' salaries range from £30 to £35 per annum. Certificated trained nurses, while employed on the private nursing staff, receive in addition to the above scale of payment an extra £5 per annum. Nurses on the private nursing staff also receive one-half of the extra charge of 10s. 6d. per week made for each infectious or massage case nursed by them. Indoor uniform, consisting of three print dresses, eight aprons, and three caps, is provided by the hospital for all the nursing staff. Pensions of £25 a year are made to nurses who, being over 50 years of age, have served the infirmary for not less than 25 years, and are unfit for further duty. Pensions are, under certain circumstances, also granted to nurses disabled after not less than ten years' service. Promotion to the position of ward sister depends upon individual merit, a necessary qualification being a three years' certificate. No night duty is required of probationers in their first year; it is usually taken for from three to six months at a time. No "special" or paying probationers are received at the Manchester Royal Infirmary.

### Hours on and Off Duty.

Probationers' hours on duty in the wards are between 7 a.m. and 9 p.m.; two hours are allowed daily for recreation, with time for meals. One day's leave is given each month, and all nurses have extra time off on Sundays. Staff nurses are on duty between 7 a.m. and 10 p.m., with time for meals, and four hours' leave on alternate days. One day's leave monthly is allowed. Sisters' hours are the same, except that they do not come on duty in the morning till 8.30. On the monthly "days off" the nurses are on duty in their wards till 9 a.m. Seventeen days' holiday is given yearly to probationers and nurses; sisters, three weeks.

### Meals.

No "food allowances" of butter, sugar, &c., are made to the nurses. All meals are served in the dining-room, with the exception of the early morning lunch, which is taken in the ward kitchens. The hours for the day nurses' meals are as follows: Breakfast, 7.40 a.m.; lunch, 9 a.m.; dinner, 1 and 1.45 p.m; tea, 4 and 4.30; supper, 8.30 and 9 p.m.

### NORTHAMPTON GENERAL INFIRMARY (163 Beds).

### Terms of Training.

Candidates for training at the Northampton General Infirmary must be between 21 and 35 years of age, and are received for one month's trial before acceptance. If found suitable, they enter the hospital on a three years' engagement, on completing which a certificate is granted. No salary is given to probationers during their first year, but they are presented with £5 at the end of the twelve months; they receive £14 during the second year, and £18 during the third year. Laundry and indoor uniform are provided by the hospital. Sisters' salaries begin at £28 per annum, rising afterwards to £30. Pupil nurses, who are received for training for periods of not less than six months, pay a premium of £20 yearly, or £5 per quarter. They provide their own uniform. Lectures are given to the nurses during their training by the house surgeon and the superintendent of nurses. Efficiency and three years' training are the essential qualifications for promotion to the charge of a ward. There is a permanent night superintendent, and probationers take night duty for three months at a time.

### Hours On and Off Duty.

Nurses are on duty between 7.30 a.m. and 9 p.m., the whole staff having two hours off duty each day, and a half day's leave every other Sunday. Head nurses are also given a half-day weekly, and probationers a day each month. Night nurses have a night off once a month, from 10.30 a.m. one day to 2 p.m. the next. Two weeks' annual leave is allowed yearly to probationers. Head nurses have three weeks' holiday in the year. Nurses are on duty until 10 a.m. on their days off. Night nurses are on duty from 9 p.m. till 9.30 a.m., and are expected to be in bed at 1 p.m.

### Meals.

All meals are served in the nurses' dining room, except the night nurses' midnight meal. Butter is given out in weekly allowances, but no other provisions save the tea and sugar for night nurses. The day staff breakfast at 7 a.m., dinner is at 12.45 and 1.15 p.m., tea at 4 and 4.30, and supper at 9 p.m. Night nurses breakfast at 8.30 p.m., and their dinner is at 10 a.m.

### For Reading to the Sick.

"THE Great Reward."

**Verses.**

Love and believe! for works will follow spontaneous,
Even as day does the sun; the right from the good is an offspring.
Love in a bodily shape; and Christian works are no more than
Animate love and faith, as flowers are the animate springtide.
Works do follow us all unto God; there stand and bear witness,
Not what they seemed—but what they were only.

—*Longfellow.*

And in despair I bowed my head:
"I have no place on earth," I said;
"For hate is strong,
And mocks the song
Of peace on earth, goodwill to men."

Then peeled the bells more loud and deep:
"God is not dead, nor does He sleep;
The wrong shall fail,
The right prevail,
With peace on earth, goodwill to men."

—*Longfellow.*

He always wins who sides with God;
To him no chance is lost;
God's will is sweetest to him when
It triumphs at his cost.
Ill that He blesses is our good,
And unblest good is ill;
And all is right that seems most wrong
If it be His sweet will. —*Faber.*

Thy prayer shall be fulfilled—but how?
His thoughts are not as thine;
While thou would'st only weep and bow,
He saith, "Arise and shine!"
Thy thoughts were all of grief and night,
But His of boundless joy and light.

Thy Father reigns supreme above;
The glory of His Name
Is Grace and Wisdom, Truth and Love,
His will must be the same.
And hast asked all joys in one,
In whispering forth, "Thy will be done."

**Reading.**

"In keeping them there is great reward."—*Ps.* xix. 11.

Not because I keep them I shall have a great reward; but in keeping them there is great reward. God Himself wants us to keep them because He loves us. He says, "O that there were such an heart in them, that they should fear Me and keep all My commandments always, that it might be well with them!" This reward is an indisputable, though too often not fully recognised fact of every Christian's experience. That we may have to keep His commandments in the very teeth of trial, loss, opposition, or distress does not touch the matter; for nevertheless, not afterward, but in the keeping of His words, He takes care to keep His word that there shall be great reward. . . . Now if we have only been obeying in mere form and letter, because we were afraid to disobey, this is not the heart-obedience which is always crowned with blessing. So if we cannot quite set to our seal that God is true to this promise, let us be quite sure that it is because we have not fulfilled His condition. And let us now, at once, ask Him to write His laws in our heart, and so to shed abroad His love in us by the Holy Ghost, that we may begin at once to keep them for very love of our glorious Lawgiver and Mediator. Then we shall know for ourselves they are not grievous, but that they are "for our good always."—*F. R. H.*

"Blessed is the man that feareth the Lord, that delighteth greatly in His commandments."—*Ps.* cxii.

### The Book World for Women and Nurses.

[We invite Correspondence, Criticism, Enquiries, and Notes on Books likely to interest Women and Nurses. Address, Editor, THE HOSPITAL (Nurses' Book World), 28 & 29, Southampton Street, Strand, London, W.C.]

WHERE TWO WORLDS MEET. By ROSA MARCH. (London: Skeffington. 1898.)

IN the compass of a neat little volume, Miss March has collected together ten thoughtful essays, none of which are lengthily worded, and which suggest, for the most part, "certain aspects of truth, which at first the writer was herself, she tells us, unable to understand, and which therefore became subjects of reflection." The words of the writer will appeal to readers who are confined to a sick room, since they emanate themselves from the chamber of a sister sufferer, and from one who, being debarred through ill-health from the distractions of a worldly life, has given her mind to those things which are above the world. In her preface Miss March writes: "As the years pass on, the spiritual world becomes more real than the fashion of this present world, which perisheth, so 'the spiritual language' is heard more distinctly by the soul, and a vision of 'the spiritual light,' of which St. John speaks, may be granted in compensation for some loss of earthly sunshine." The writer's pages bear evidence of a thoughtful mind, and, among others, comprise the following subjects: "Spiritual Language," "Spiritual Light," "True Riches," "Thoughts on my Way to Rest."

SHORT SERMONS FOR SICK ROOMS. By the Rev. JOSIAH BATEMAN, M.A. (Charles Thynne, Wycliffe House, Great Queen Street, E.C.)

A NEW edition of the above volume by the late Rev. Josiah Bateman has lately been issued, the price of which is 1s. 6d. nett. The sermons are really what they profess to be, "short sermons" for "sick rooms," and were written, as Mr. Bateman tells us in his preface, at the request of a near and dear relative, who was confined to her room for many weeks with depressing and dangerous illness. The first editions of these excellent little sermons appeared about 1870, and it proves how acceptable they have been when a third edition is called for now, and we would call the attention of the sick to this helpful little volume now produced in a handy size, and set up in large type for the convenience of reading in a sick bed.

MEMOIRS OF A YOUNG SURGEON. By FREDERICK ASHURST, M.D. (London: Digby Long and Co.)

THE "Memoirs of a Young Surgeon" comprise six stories, short ones, written with a view to entertaining the reader. As an illustration of this, we give from the first little story the following quotation: "How true it is that a patient's gratitude is part of his disease, and is most declared when the fever is highest, cools off during convalescence, and entirely disappears with the return of health." This was recognised by Cordus in the sixteenth century, for a translation of his epigram reads like this:—

"The Physician like an angel seems,
When by our bed he brightly gleams;
And like unto a God is he,
When he has removed the malady.
But in a different light we view
The Doctor when his bill is due;
Our altered eyes we at him level,
As though he were the very devil."

### The National Union of Women Workers.

### ANNUAL CONFERENCE AT NORWICH—(*continued*).

### WORK AMONG MEN AND BOYS.

DEBATES on the subjects of "Our Workhouse Boys" and "The Difficulties of Working Clubs and Classes for Men and Boys" took place in another hall during Tuesday afternoon, when Mrs. Eaton Lascelles related her experiences amongst her workhouse boys, and Miss Violet Brooke-Hunt urged the teaching of handicrafts to men and boys through the medium of clubs and classes.

### THE FRIENDLY SOCIETY MOVEMENT.

Mrs. Peile, of Cambridge, wife of the Master of Christ's College, treated the subject of the Friendly Society Movement among women from "The Individual Point of View" on Tuesday evening, her paper being followed by one dealing with it from the "Economic and Trade Standpoint" by Miss Edith Deverell, of the London School of Economics. Mrs. Peile spoke of the great benefits to be derived by the individual woman in belonging to such societies, benefits material, educational, and moral. She traced the history of the movement, noting the rapid recent increase of female lodges, and touching on the vexed subject of mixed courts, which in her view presented considerable difficulties in the adjustment of the different payments for men and women.

Miss DEVERELL said that a great number of women's thrift societies appeared to exist, as shown by recent investigation, the number of women belonging to these smaller clubs exceeding those attached to any of the great recognised orders. These small clubs were useful, but the educational value of membership was undoubtedly less than in the case of the great friendly societies. Miss Worsley (of the United Sisters' Society), Miss Edith Page, Miss Collett, and Miss Brodie Hall continued the discussion, the question of maternity allowances, introduced by Miss Page in the course of her excellent speech, causing some animation, Miss Collett asking, "Should not men be asked to take their share of maternity allowances?" to which Miss Page replied that women would do well not to wait for that possibility.

### CARE AND NURSING OF THE EPILEPTIC.

Miss GIBSON, matron of the Birmingham Infirmary, who read the first paper on the morning of Wednesday, the 26th ult., dealing with "The Care and Nursing of the Epileptic," was listened to with deep attention and interest. Miss Gibson began by expressing her astonishment that so little had hitherto been done for the more than 30,000 epileptics in this country, and that there were so many theoretical and so few practical sympathisers with them. She pointed out the disabilities under which every epileptic suffered, whether amongst the poor or the rich, in the one case too often from neglect, and in the other from an almost exaggerated tenderness which defeated its own object and added to the nervous irritability of the patient. People had an extraordinary horror of fits; the epileptic was debarred from school or college, and in the case of the poorer classes, too often drifted into imbecility and the workhouse; employment was almost impossible to find by those still able to work, and since the passing of the Workmen's Compensation Act, employers had in many cases discharged epileptic work-people, because they would not undertake the responsibility of too possible accidents. Miss Gibson urged that more should be done to make the possibilities of epileptics equal to those of the sound in health, especially in the increase of the colony" "system, which, in her view, gave the best possible results. There were only three homes of this kind in England—the Maghull Homes, the Chalfont Colony, and Lady Meath's Home at Godalming—of each of which she gave some details speaking of the need for more accommodation for better class patients, and of their importance as nurseries for the trained and skilled attendant. Miss Gibson spoke of the special qualifications needed for the nursing of these cases, instancing the extraordinary success achieved by a nurse who had once worked under her, and whose observation became so keen that she was almost always able to recognise early signs of an approaching attack, and could frequently avert a fit by careful avoidance of irritating causes. Neither kudos, red crosses, nor medals were to be gained by those who devoted their lives to the care of the epileptic, but she commended the work to those possessed of the somewhat rare qualifications for it, adding that "the quietest work is often the best."

Miss ANNIE CAZENOVE, hon. secretary of the Meath Home, opened the discussion which followed, and spoke of the willingness with which Guardians would pay for placing patients in the homes provided, of the awakening which she hoped was taking place in England on the whole subject, and of the extraordinarily successful results which followed proper treatment of epileptic patients.

Mrs. BASIL HOLMES reported on the mixing of epileptic in Ireland with the ordinary inmates of the workhouse, and Miss PEARCE, Miss MARY SIMMONDS, and Mrs. PERCY BUNTING took part in the discussion, the latter lady laying special stress on the fact that much could be done for epileptics who were not bad enough for entire removal from their own homes, and who needed a kindly helping hand to enable them to continue earning their living so long as they were harmless to themselves and others, and competent to work.

### CARE AND TRAINING OF DEAF MUTES.

Mrs. HENRY WARE, President of the Ladies' Auxiliary of the British Deaf and Dumb Association, and Miss BOULTBEE were the chief speakers on this subject, Mrs. Ware pleading for more workers and more money for the establishment of special missions amongst the deaf and dumb, and Miss Boultbee, who is herself a teacher of the oral system, giving instances of the great advantages offered by it, and of the necessity of developing in this manner the intelligence of the children, who could thus be made to "listen with their eyes," and to whom learning to talk need present no great difficulty.

### CARE AND TRAINING OF THE FEEBLE-MINDED.

Following on this paper came one by Miss GRAYSON, of Liverpool, on "The Care and Training of the Feeble-minded" in which she spoke of the necessity for differentiating between idiots, imbeciles, and the "feeble-minded," recommending the establishment of special homes for the latter, and the need for combined action amongst those interested in the subject.

Mrs. BURGWIN said that the question was a national one, but in this, as in so many instances, voluntary work might lead the way, and it was most important that the right lines should be established before legislation followed. Mrs. Burgwin gave interesting instances from her own experience of how much might be done for feeble-minded boys and girls, and spoke of the need for dealing with children who were too feeble for Board School classes, yet were at large, a danger to themselves and others. Such should be treated in "custodial homes," but not as paupers. Mrs. Bracey, Mrs. Washington Palmer, and several other ladies spoke on this subject.

### TWO WORKHOUSE QUESTIONS.

On Wednesday afternoon the Conference set itself to consider two questions especially interesting to women who act as guardians of the poor, of whom there were a number present. These were: "The Treatment of Parents o

Illegitimate Children in Workhouses," and "Children in Need of Legal Protection."

Mrs. HEALEY, whose paper on the first subject was a wise and helpful one, laid special stress on the fact "that it is a one-sided effort, and unfair to the child, that only seeks to redeem the woman, leaving her with the great problem of how to support her child unsolved. A great effort must be made to reach the father, and oblige him to bear some of the penalty."

Miss CLIFFORD urged more investigation into each case; she spoke of the necessity of "drawing," not "driving," and yet, while trying to alleviate the wrong, of taking no steps that might increase the cause of it.

The second paper was read by Mrs. BARNETT, who pointed out the pressing need for legislation to deal with children who pass "in and out" of the casual wards, the workhouses, and the workhouse schools. She urged her audience to give their support to the Bill dealing with this matter which had been promoted by the State Children's Aid Association, and was introduced last year by Mr. Ernest Flower, M.P., but was crowded out in the rush of other measures.

Many saddening details of these present evils were dwelt on by various speakers on both papers, and following on the somewhat depressing discussion of the morning, the spirits of members were at a low ebb. The delightful surprise in store when suddenly, the speeches over, Mrs. Booth raised her hand for silence, and Madame Antoinette Stirling's beautiful voice filled the hall with the comforting notes of "O, Rest in the Lord," was therefore all the more appreciated.

### HOME WORK.

Wednesday's programme was a very full one. Whilst the above-mentioned papers were being discussed, another meeting "for young ladies" was held in Noverre's Rooms under Lady Battersea's presidency, and at tea time an informal meeting gathered at the School of Cookery in connection with the Women's Local Government Society. In the evening the members of the Conference met again to consider the subject of "Home Work as it affects (*a*) Women and (*b*) Children."

Miss MARGARET IRWIN, secretary of the Glasgow Council for Women's Trades, who has done much for the cause of labour in Scotland, read the first paper, in which she gave details of the terrible condition under which women worked at various trades in their own miserable homes, and urged that such homes should be under legal inspection, and that the extra work taken home by workshop hands to finish after hours should be entirely prohibited.

Mrs. HOGG's paper on "Home Industry and its Bearing on Child Life" was delivered with so much force and feeling that her hearers were thrilled with horror at the vivid picture brought before them of the evils attending this unsupervised system of child labour. Mrs. Hogg showed how the stringent factory regulations had had the effect of driving the work out of the factories into the homes of the people, where children are employed for far longer hours with no supervision whatever. Children attending school were forced to toil in every spare moment, often till late into the night, in aiding their parents to add a few pence weekly to the family income. Mrs. Hogg stated that the Women's Industrial Council intended to bring a Bill into the House of Commons next session to ameliorate the present state of things.

In the discussion which followed, Lady KNIGHTLEY OF FAWSLEY and Miss HEATHER-BIGG had something to say on the other side of the question, deprecating too much compulsory interference with the freedom of home life; but the eeling of the meeting was undoubtedly with the first speakers, and Mrs. HICK's words, coming with special meaning from one herself, a "working woman," met with much applause. Mrs. REEVES gave a cheering account of what legislation had done on this matter in New Zealand, and other speakers followed.

### THE LAST DAY'S MEETINGS.

The annual meeting of the National Council of Women filled most of the time on Thursday, the last day of the Conference. Mrs. ALFRED BOOTH was re-elected president for the forthcoming year, and the election of the executive body was also proceeded with. The programme for the International Congress which is to take place next June came up for discussion, and after this the hall filled to overflowing with those who wished to hear Lady BATTERSEA's paper on "The Amenities of Life." It was an excellent paper, and the hints given therein on the "beauty of beautiful manners" and on "amenities" generally, both in public and in private life, were well worth listening to. A paper from Miss LIDGETT on "An Ideal, a Practical Need" came next, and Mrs. BOOTH's concluding presidential address, followed by very cordial votes of thanks to the various members of the Norwich Committee, brought the business of the Conference to a successful close. A meeting of Rescue Workers was held on Friday morning.

### The Temperance Male and Female Nurses' Co-operation.

THIS is a new venture, opened in January at 45, Beaumont Street, which is already meeting with considerable success. It is a private registry for trained nurses who have, besides the full training qualifications, that of being total abstainers. The secretary, Mr. G. Gordon, has been a nurse for upwards of 20 years, and has a good connection amongst well-known medical men; the lady superintendent is his wife. The female nurses must have a certificate of three years' training at a general hospital, and the male nurses hold the certificate of the Medico-Psychological Society. They receive their own fees, which are from one and a half guineas upwards, and pay a definite percentage on all cases to the home. There is residential accommodation on the premises, and nurses may board there if they wish at their own cost. A code of rules, arranging the nurses' hours of duty, expenses, recreation, and rest, are sent to each patient, with a request that a report of the nurse's conduct may be returned at the conclusion of his or her attendance.

### Novelties for Nurses.

### WARM WINTER GARMENTS.

EVERYONE is now renewing their stock of winter garments, and the main qualities to be sought are warmth, comfort, and durability. All these are to be found in the admirable stockingette garments manufactured by the Hermes Stockingette Company, of 77, Fore Street, E.C. Nothing could be more cosy than the Hermes dressing gown, a most inexpensive and attractive garment, whilst the Hermes Matinée, which is in effect a tasteful blouse, is ideally comfortable. The stockingette material used in all the garments is of a soft and silking finish, which could not irritate the most sensitive skin, and its extreme elasticity enables a perfect fit to be secured without pressure. The Stockingette Company supply every class of garment to which the material is applicable, from stockings to divided skirts, dressing gowns, &c. All are beautifully finished.

### Everybody's Opinion.

[Correspondence on all subjects is invited, but we cannot in any way be responsible for the opinions expressed by our correspondents. No communication can be entertained if the name and address of the correspondent is not given, as a guarantee of good faith but not necessarily for publication, or unless one side of the paper only is written on.]

### HOSPITAL HOUSEKEEPING.

"A SYMPATHISER" writes: "The old order changeth, giving place to the new." This is a very good saying, especially when it can be applied to hospital housekeeping, for it will cheer the hearts alike of patients and nurses. So numerous and important are the improvements that have been made in many hospitals, that it makes one ask, "Why cannot it be possible for the same advantages to be provided in all our hospitals?" Dealing with the subject in an August number of THE HOSPITAL, "A Late Matron" writes very strongly, but not without cause, and it is a pity that no present matron has seemed sufficiently interested in the subject to write a reply. From my own experience of hospital management I can confirm the statements made by "A Late Matron" in regard to many particulars. Why, I would like to know, is cod served up with such monotonous regularity as is the case? One would suppose that those responsible are unaware that there is other fish in the sea, and quite as cheap; but I suppose it has been the rule in these hospitals to supply cod, and like a good many other regulations which could be named, it must not be altered. But just fancy boiled cod, dubbed "an extra," being given to patients who are really ill, and not merely once or twice, but day after day for months. Cod, at the best, is rather an insipid dish, and what must it seem to the poor patient whose palate requires, or should require, a little humoring. Then, again, in many institutions, rice pudding, to my knowledge, is regarded as the only one pudding (I suppose the patient's safety would be jeopardised if any other were allowed) which will restore the sufferer to a condition of convalescence. The system is more than a senseless one—it is wrong. It must retard the patient's recovery, while in the case of the nurses the hateful monotony of always being served with one kind of pudding, fish, and jam, &c., amounts to a refined torture which must produce an ill effect upon the nurses, and, consequently, upon the proper performance of their important duties. The dietary in general in a good many hospitals requires overhauling. While it remains as at present there is no wonder that so many girls who wish to become nurses cannot stand the work, although, of course, the true cause of their failure is never acknowledged by those in authority. To those acquainted with the inner working of a good many of our hospitals it is a difficult matter, when the position and the rank of nurses are spoken about, not to smile or indulge in some sarcastic comment. An indispensable qualification which lady nurses must possess is the ability to perform any amount of work without grumbling, and to appreciate a dietary which an agricultural labourer would turn his nose up at.

### THE POOR LAW NURSE.

"A SUPERINTENDENT OF NURSES" writes: I agree with your correspondent that it is felt to be a real grievance for trained and untrained nurses to work for the same salary. I had a promising young nurse last winter fresh from her hospital of fifty beds with a three years' certificate and a vast idea of her own importance; she left chiefly because she could not brook this condition of things. Had she had patience and worked well for six months she would now be in charge of wards, she being the only assistant with a certificate, of the value of which boards of guardians have a very fair estimate. The Poor Law is not the only branch of nursing where partly-trained women obtain the same work and pay as their fully-qualified sisters. I do not think much of the trained nurse who is not certificated. I have seen many of them; there is generally a plausible tale of a serious illness or home duties, but I have (with one exception) come to the conclusion either that their training school wisely rejected them or that they left to escape its discipline; that such should form nursing homes and be sent to acute cases is a scandal. We need something like a trades' union to stop it, and also to instruct nurses as to the market value of their training and the L.O.S. certificates, experience, age, &c., and I would forbid their taking less. I am running away from my subject. I would suggest that when only untrained women apply in answer to an advertisement for a trained nurse, the difference should be pointed out to them, and the post offered at, say, £5 less salary, or both sums might be mentioned in the advertisement. And here I may say that though the Poor Law have the longest advertisements, they really give the least information of any in your columns, being specimens of the useless routine of the system, so that a nurse has often to take up her duties before she learns what is required of her, even to the number of beds and nurses. I want to know what to do with the Mrs. Gamp of the Poor Law; I have met her species—she is large, she knows her rights (and gets them), she makes a cup of tea when so disposed, she keeps her wards clean, and rules her people with a rod of iron. She is innocent of antiseptics, lays down dressings anywhere,; yet succeeds with "legs." She secretly thinks scorn of general orders and evades them cunningly. She is an inveterate gossip. In contrast to her is the nurse who has had several years' private work, who is kind to the individual patient, and works hard all day long, yet her wards are never in order, contraband articles are eaten under her very nose; nothing is done at the right time. Give me untrained women, about thirty, with strong arms, willing hearts, and clear heads, and I will make something of them for the Poor Law, but I do not consider the training good for anything else except as attendants on the chronic invalids, and monthly nurses.

### Opening and Benediction of St. John's, Deptford.

IN one of the many squalid streets of Deptford the Nursing Sisters of St. John have built a convenient and picturesque home for their workers in this neighbourhood. It was opened on November 1st, 1898 (All Saints' Day) by the Bishop of Rochester, who is the visitor to the community. The Bishop, accompanied by his chaplain, the Rural Dean and the Rector of the parish, visited the sisters'-room, dining-room, kitchen, pharmacy, and dormitories in succession, offering a short prayer, to which a suitable response was given by those present, in each, until the conclusion of the service, which took place in the oratory. On the ground floor is the sisters' sitting-room, and opposite it a similar room used by all as a dining-room, and by the nurses as a sitting-room. A side entrance opens into a small waiting hall, out of which is the pharmacy, where patients have their ailments seen to, whilst a small room beyond is used as an office and for private interviews. There is a lavatory conveniently placed for the patients' use. The kitchens are large, light, and airy. On the floor above are four bed-rooms, two bath-rooms, lavatories, and a small oratory. These rooms are for the use of parish workers or lady pupils, the sleeping accommodation for the staff nurses and servants being on the floor immediately above. The walls are distempered in deep cream colour, with dadoes of a soft blue. Most of the floors are of polished wood, on which one or two mats are spread for comfort sake. The lighting is by gas; the stoves are of the modern slow-combustion type. Outside is a shed for a wheel chair, and a tiny garden, which promises, nevertheless, to be pretty. The cost of the home was defrayed by an anonymous donor, who gave £5,000 in 1894 for the home and work. The freehold site was bought for £500, and the building cost £1,806. It is arranged that two sisters and a staff of nurses shall live in the home, and nurse the sick poor around them, of whom such as are able will come to the pharmacy and have their wounds dressed, and so save the cost and worry of a journey to London.

### Appointments.

ALBERT INFIRMARY, WINSFORD, CHESHIRE.—Miss A. Wetherell has been appointed Matron of this infirmary. Miss Wetherell was trained at the Nightingale School, St. Thomas's Hospital. She then acted as staff nurse at St. Marylebone Infirmary, and afterwards as head nurse at Darlington Hospital. She has been for two years sister at the Royal Albert Edward Infirmary, Wigan, and also home sister of the Salop Infirmary, Shrewsbury.

THE GROSVENOR HOSPITAL FOR WOMEN AND CHILDREN, VINCENT SQUARE, WESTMINSTER.—Miss Mary F. May, who was trained at St. Bartholomew's Hospital, and who has been staff nurse there since 189 7, was appointed Lady Superintendent of the above hospital on the 2nd inst.

BIRMINGHAM AND MIDLAND EYE HOSPITAL, BIRMINGHAM.—Miss A. H. Marriott was appointed on the 7th inst. Matron of this institution. Miss Marriott has been assistant matron at the General Infirmary, Birmingham.

EYE, EAR, AND THROAT HOSPITAL FOR SHROPSHIRE AND WALES.—Miss J. Allen has been appointed Matron of this hospital, where she has been trained, and has been charge nurse for 10 years.

### Presentations.

NURSE DAVIS, who had belonged to the Trained Nurse's Institute, Weymouth, for three years, was presented, on leaving to be married, with a very handsome clock from the superintendent and nurses. She also received many presents from patients and friends, including a silver cruet stand, silver teapot, brass inkstand and candlesticks, a gold and pearl brooch, and many other useful and valuable presents. Nurse Davis obtained the usual certificate given at the end of three years' training.

ON the occasion of Miss Burton's marriage with Dr. Legge, Superintendent of the Derby County Asylum, she was presented with a gold and pearl necklet by the officers and staff of the Fife and Kinross Asylum, of which institution she was matron. The marriage took place on the 2nd inst., and the presentation was made on October.

### Notes and Queries.

**The contents of the Editor's Letter-box have now reached such unwieldy proportions that it has become necessary to establish a hard and fast rule regarding Answers to Correspondents. In future, all questions requiring replies will continue to be answered in this column without any fee. If an answer is required by letter, a fee of half-a-crown must be enclosed with the note containing the enquiry. We are always pleased to help our numerous correspondents to the fullest extent, and we can trust them to sympathise in the overwhelming amount of writing which makes the new rules a necessity.**

**Every communication must be accompanied by the writer's name and address, otherwise it will receive no attention.**

*London Nursing Association.*

(70) I am anxious to join a London co-operative association for nurses. Would you kindly let me know how to obtain a list of these agencies, or what steps should I take to start nursing on my own account?—*A. L.*

There is only one Nurses' Co-operation of Nurses for nurses, that at 8, New Cavendish Street, W. "Burdett's Official Nursing Directory," price 5s., from the Scientific Press, gives full particulars of the other more important nursing associations. Work up a connection with medical men first; the rest follows.

*Completion of Training.*

(71) Having had twelve months' training, I wish to become assistant nurse in a general hospital where I could gain a three years' certificate?—*Nurse Crawford.*

Your best plan would be to advertise. Such certificates are not usually granted.

*Leper Hospitals.*

(72) Would you be so kind as to give me some information with regard to hospitals for nursing cases of leprosy? Are there any in Europe, and which are the chief ones abroad? Could you tell me the geographical name of the "leper island" somewhere near Samoa?—*Bee.*

There are such a number of leper hospitals, that we must refer you to "Burdett's Hospitals and Charities" (5s., The Scientific Press, London).

*Honolulu.*

(73) Could you tell me how to get information as to nursing in Honolulu, both private and hospital?—*N. B.*

Possibly Miss Louisa Beale, the Matron of the Colonial Hospital, Suva, Fiji, would give you information if you wrote and asked her.

*Free Training.*

(74) Are there any free institutions where poor girls are trained to be hospital nurses?—*Ebor.*

Most hospitals accept poor candidates if they are suitable. Apply to the matron of your nearest large infirmary or hospital.

*Cheap Homes.*

(75) Could you give me the address of a convalescent home at Ventnor or Bournemouth for a patient who is able to pay a small weekly fee?—*H. C.*

"Burdett's Hospitals and Charities" (Scientific Press, London, 5s.) gives a very comprehensive list, and there is a list of cheap holiday homes and homes of rest to be obtained from Miss F. E. Cole, 188, Ebury Street, S.W., price 1d.

*Argentine Republic.*

(76) I would like to know something of the hospitals in the Argentine Republic, and if it would be advisable for nurses with three years' training to go there?—*Argentine.*

"Burdett's Hospitals and Charities" (price 5s., from the Scientific Press, London) gives a list of hospitals. Will any of our readers help "Argentine"?

*Medico-Psychological Certificates.*

(77) Would you inform me if it is possible to obtain the certificate of the Psychological Association without entering a county asylum for that purpose? The medical officer of this workhouse is willing to give us the necessary lectures. I have had about twelve years' experience with the insane—five years in an asylum, the remainder in workhouses and private work.—*T. J.*

Write direct to the Registrar, the Medico-Psychological Society, 11, Chandos Street, W.

*Indoor Gymnastics.*

(78) I should be very grateful to you if you would be so kind as to tell me through your paper where I can get particulars about Dowd's apparatus for indoor gymnastics, an arrangement that can be fixed on a bedroom door. I have seen it advertised many times, but just now that I want it I cannot find the address of the makers anywhere.—*Exerciser.*

We send you particulars by post of the Household Physical Trainer, 20s. net, Sandow's Combined Developer, 12s. 6d. The address of Dowd's Health Exerciser is: Scientific Physical Culture School, Prestbury Road, Macclesfield.

*The Cost of the L.O.S.*

(79) What would the cost of training as a monthly nurse be at Queen Charlotte's Hospital, to one who has no general training? Are there other training schools as good? and could one get the L.O.S. certificate at them?—*A. L. M.*

There are many good and nearly as well-known training schools for midwifery nurses as Queen Charlotte's. The fees, though varying at each institution, are the same to all, trained or untrained. Fees at Queen Charlotte's are 15 guineas for six weeks' monthly training, and £26 5s. for L.O.S.; the cost of uniform, laundress and examination fees is borne by pupil. The fees at the East-end Mothers' Home are the cheapest for the three months' training for the L.O.S., they only amount to £15 15s.; extras as at Queen Charlotte's.

*District Nursing.*

(80) Can you tell me the best way to procure a list of parishes where district nurses are engaged nursing the poor, and where a small charge is made for maternity nursing?—*District Nurse.*

Apply to the Secretary, Queen Victoria's Jubilee Institute for Nurses; the Queen Katherine's Hospital, Regent's Park, W.; and to the Secretary, the Ockley Nursing Association, The Cottage, Ockley.

*Short Maternity Training.*

(81) Can you inform me of a hospital where I can get one month's training in midwifery?—*F. E. W.*

The shortest time for training a monthly nurse is six weeks: for a midwife three months. Both periods much too short for other than nurses already trained. See other queries on the subject

*Maternity Work.*

(82) Kindly inform me whether there is a co-operation where a nurse with maternity certificate only could get work; if not, how is the best plan to start work?—*E. E.*

There are sometimes advertisements in our columns for maternity nurses only, inserted by institutions conducted on a co-operative plan. To get work otherwise a nurse must either advertise or have a private connection.

### ANSWERS REQUESTED.

*Etiquette amongst Nurses.*

(83) Night Superintendent wishes to know (1) if it is usual for an assistant nurse, coming on duty for the first time, to inform her that the matron said she was to go on duty in a certain ward? Is the N.S. within her rights if she refuses to take an order given in such an informal way? (2) Should a N. S. be informed beforehand when night nurses are taken off and put on duty? (3) Is it usual for the N. S. not to be allowed to know when the nurses under her have a night off duty or shorter leave; and is it not the custom in some hospitals for the N. S. to arrange the leave for the nurses under her? (4) Is it not the custom for the N. S. to make a verbal report to the Matron concerning the nurses at least once in 24 hours?

